# Solvent Dependency of the UV-Vis Spectrum of Indenoisoquinolines: Role of Keto-Oxygens as Polarity Interaction Probes

**DOI:** 10.1371/journal.pone.0073881

**Published:** 2013-09-26

**Authors:** Andrea Coletta, Silvia Castelli, Giovanni Chillemi, Nico Sanna, Mark Cushman, Yves Pommier, Alessandro Desideri

**Affiliations:** 1 Dipartimento di Biologia, Università degli Studi di Roma “Tor Vergata”, Roma, Italy; 2 Consorzio interuniversitario per le Applicazioni del Supercalcolo Per Università e Ricerca, Roma, Italy; 3 Department of Medicinal Chemistry and Molecular Pharmacology, School of Pharmacy and Pharmaceutical Sciences, and the Purdue Center for Cancer Research, Purdue University, West Lafayette, Indiana, United States of America; 4 Laboratory of Molecular Pharmacology, Center for Cancer Research, National Cancer Institute, Bethesda, Maryland, United States of America; University of Bologna & Italian Institute of Technology, Italy

## Abstract

Indenoisoquinolines are the most promising non-campthotecins topoisomerase IB inhibitors. We present an integrated experimental/computational investigation of the UV-Vis spectra of the IQNs parental compound (NSC314622) and two of its derivatives (NSC724998 and NSC725776) currently undergoing Phase I clinical trials. In all the three compounds a similar dependence of the relative absorption intensities at 270 nm and 290 nm on solvent polarity is found. The keto-oxygens in positions 5 and 11 of the molecular scaffold of the molecule are the principal chromophores involved in this dependence. Protic interactions on these sites are also found to give rise to absorptions at wavelength <250 nm observed in water solution, due to the stabilization of highly polarized tautomers of the molecule. These results suggest that the keto-oxygens are important polarizable groups that can act as useful interactors with the molecular receptor, providing at the same time an useful fingerprint for the monitoring of the drug binding to topoisomerase IB.

## Introduction

The description of the solvent-dependent electronic properties of drug compounds permits the identification of the preferred conformations and of the most polarizable chemical groups providing important indications on their role in the interaction with their bio-molecular targets [1], [2]. Such an approach has been followed to investigate the camptothecin family of drugs (CPTs) through a series of combined spectroscopic and computational studies that have provided from one side the diagnostic signatures of different forms of the drugs and, from the other one, the importance of the environment in the spectroscopic signal modulation, opening the way to monitor the drug-receptor interaction [3]–[5]. The camptothecin family drugs have a relevant clinical role since they are antitumor compounds that have as a unique target topoisomerase IB (Top1), an essential enzyme involved in the maintenance of genome integrity [6], [7]. Top1 is affected by the intercalation of CPT at the DNA cleavage site and the resulting stabilization of the covalent Top1-DNA complex and inhibition of DNA religation causes the collision of the single-stand break with the replication fork, leading to irreversible double-strand DNA breaks and bringing cells to death [8].

**Figure 1:**
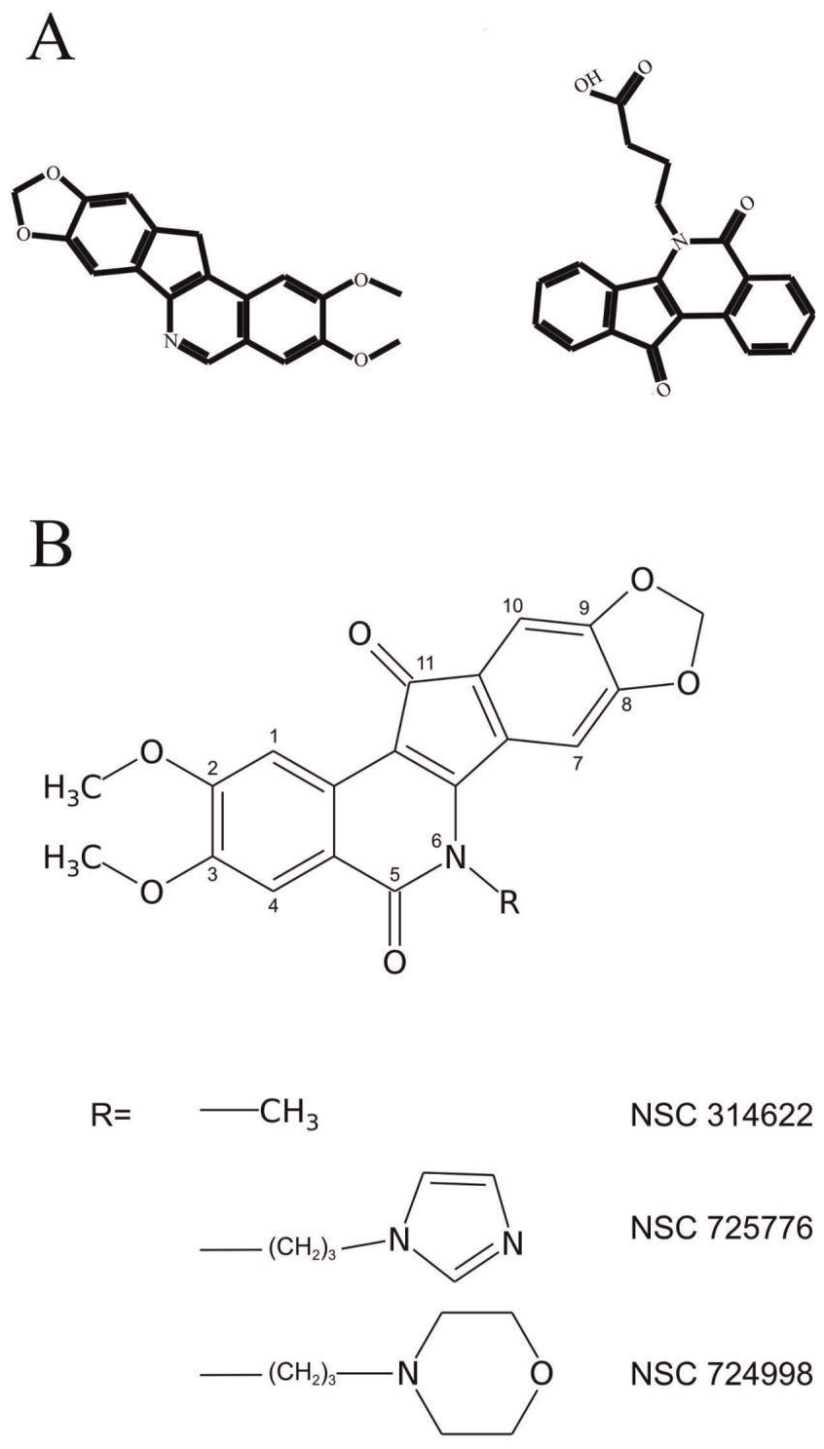
Compounds under investigation. A) Schematic representation of AI-III-52 (left) and MJ-238 (right) IQN derivatives for which the X-ray structures of the Top1-DNA-drug complex are available (PDB-ID 1TL8 and 1SC7 for AI-III-52 and MJ-238 respectively). The compounds have the same relative orientation observed in the X-ray structure B) Chemical structure of the investigated indenoisoquinolines.

The details of the interaction of the CPTs with the Top1-DNA covalent complex have been described by X-ray diffraction studies concerning the Top1-DNA-drug ternary complex [9]–[11]. The description has been expanded by the simulations of the Top1-DNA-topotecan ternary complex, which has highlighted the drug-binding effect at short and at long range, describing the stability of the drug-protein interactions and the long-distance protein comunication [12]–[16]. Although CPTs are presently the only Top1 inhibitors clinically approved for cancer therapy, they have intrinsic drawbacks such as: 1) CPTs are chemically unstable and rapidly convert to a carboxylate form that binds to plasma proteins; 2) the Top1-DNA-drug cleavage complex reverses within minutes after drug removal, requiring long infusion time of the drug; 3) CPTs are rapidly and extensively transported outside of the cell by cellular efflux pumps; 4) the side-effects of CPTs are dose-limiting and potentially severe [17]. Because of these limitations new inhibitors have been developed in the last years and among them indenoisoquinolines derivatives (IQNs) seem to be the most promising ones [6], [18]–[25]. The X-ray structures of the ternary complexes containing the IQN derivatives (AI-III-52 PDB:1TL8 [11], or MJ-238 PDB:1SC7 [10]) having a flipped orientation one respect to the other (see Fig. 1A), show an interfacial binding mechanism similar to CPTs, with the presence of hydrogen bonds with Top1 residues and a 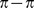 stacking interaction with DNA bases at the cleavage site. This latter property primarily accounts for the preferential orientation of the molecule respect to DNA as shown using quantum mechanical (QM) calculations [26]–[28]. QM calculations represent a powerful approach for the identification of chemical factor influencing the electronic properties of the drugs and of the chemical groups having a preferential interaction with the molecular target, i.e. the Topo1-DNA complex.

For the two IQN compounds (NSC724998 and NSC725776 in Fig. 1B) currently under Phase I clinical trials [19] and their parental compound (NSC314622 in Fig. 1B), which share the same molecular scaffold of AI-III-52 (Fig. 1A left) and the keto-oxygens in position 5 and 11 as MJ-238 (Fig. 1A right), the X-ray structures are not available, and QM calculations have not been reported.

In this paper we present an integrated computational and experimental UV-Vis investigation of the electronic and structural properties of these IQN derivative in non-polar (CCl4) polar (DMSO) and protic (PBS) environment. Our investigation permits the identification of spectral signals related to the keto-oxygens in position 5 and 11, two chemical groups that have been shown to be important in the hydrogen binding with the Arg364 residue of Topo1 and that may represent an useful signature for monitoring the drug-molecular target interaction.

## Results

### Uv-Vis Spectrum in Aprotic Solvents

**Figure 2:**
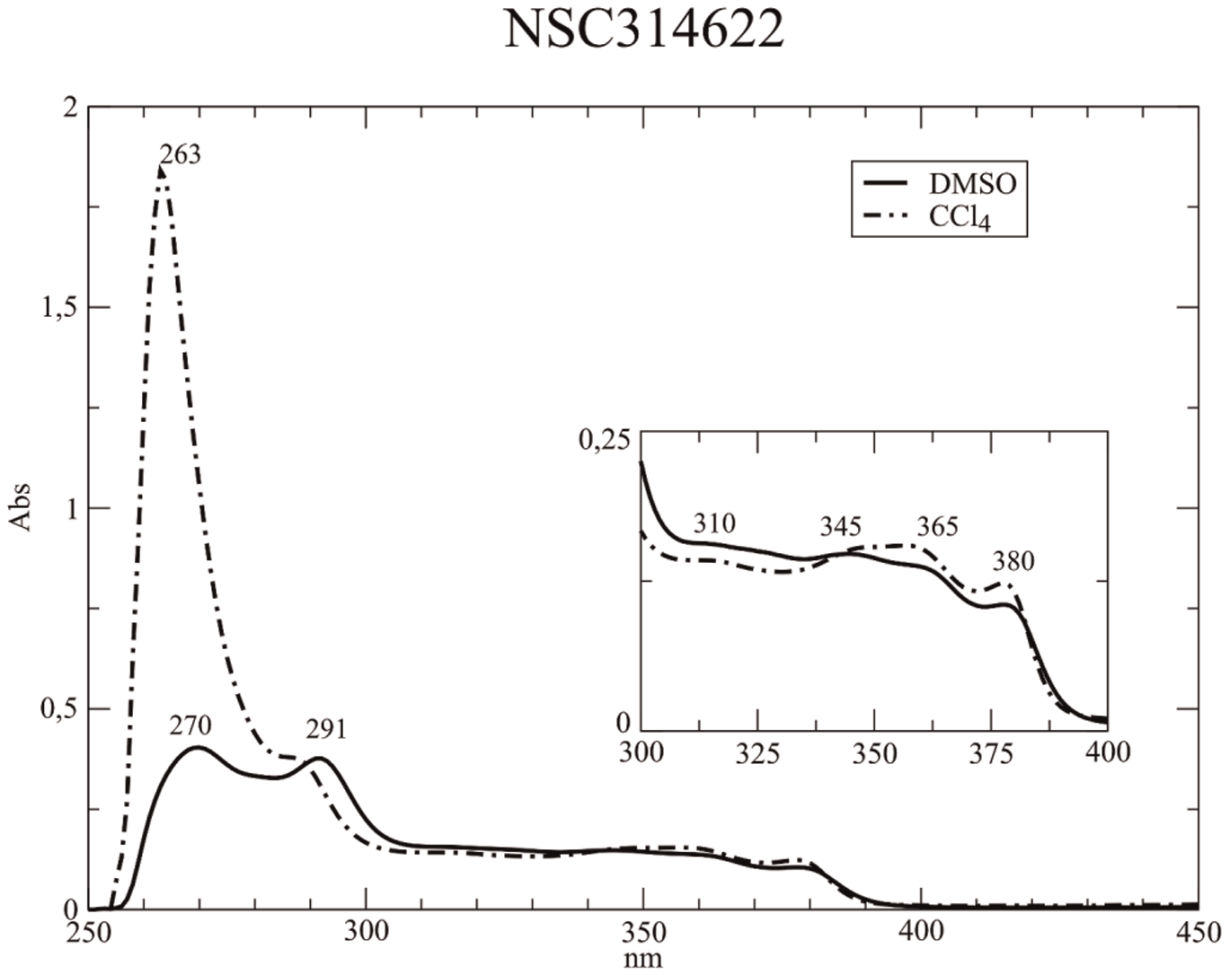
NSC314622 in aprotic solvents. UV/Vis absorption spectra of the indenoisoquinoline NSC314622 in CCl4 (dot-dashed line) and in DMSO (full line). The wavelength of the peaks are annotated. The inset represents a close-up view of the absorption in the 300–400 nm interval.

**Figure 3:**
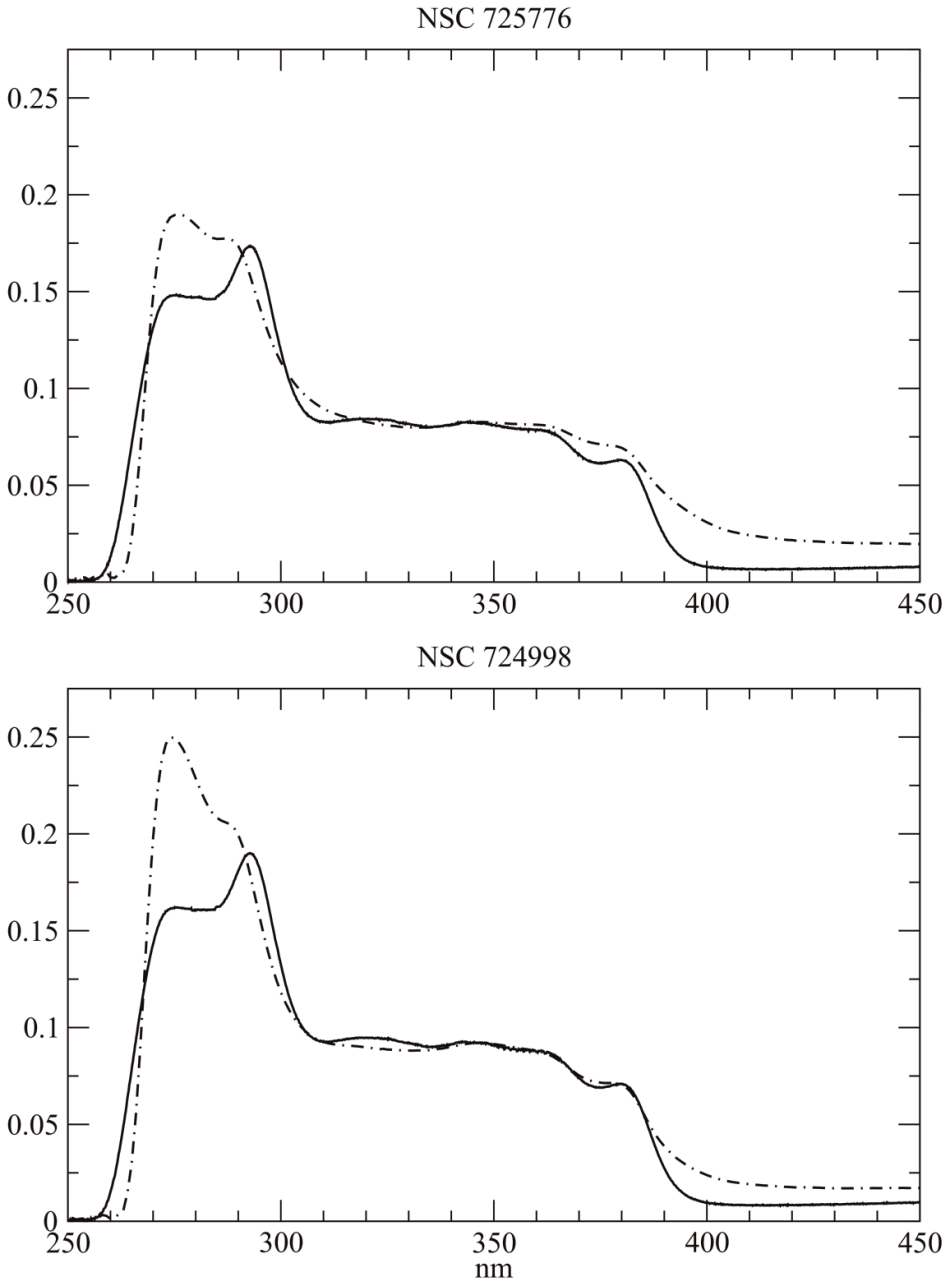
Derivative compounds in aprotic solvents. UV/Vis absorption spectra of indenoisoquinoline derivatives NSC725776 (top) and NSC724998 (bottom) recorded in CCl4 (dot-dashed lines) and in DMSO (full lines).

The experimental UV-Vis spectra of NSC314622, NSC725776 and NSC724998 (see Fig. 1B) in CCl4 and DMSO in the 250–450 nm range are shown in Fig. 2 and Fig. 3. The three compounds have an identical scaffold and differ only for the atoms on the lateral chain R that is a methyl group a 3-imidazolyl-1-prolyl group and a 4-morpholinyl-1-propyl group for NSC314622, NSC725776 and NSC724998, respectively. The general features of the spectra of the three compounds are very similar suggesting that the electronic structure of the boundary orbitals is slightly affected by the atoms in the lateral chain. A reduction of the 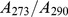, intensity ratios is observed for NSC314622 when passing from CCl4 to DMSO, and a similar decrease, although with a lower extent, is observed for the two derivatives.

In order to correlate the molecular electronic structure with the UV-Vis experimental bands positions, the geometry of the three compounds has been optimized at the B3LYP/6-31+G(d,p) level of theory in either CCl4 or DMSO, including solvent polarizability through the C-PCM method (see Materials and Methods section). The geometrical structures of the three compounds, in the two solvents, are very similar. The Root Mean Squared Distance (RMSD) between the geometrical structures of NSC314622 in CCl4 and DMSO is 0.03 Å, while between those of NSC724998 and NSC725776 the RMSDs are 0.25 Å and 0.05 Å respectively (0.05 Å and 0.03 Å taking into account only the scaffold carbon atoms), indicating that, in the two solvents, a small rearrangement occurs only for the lateral chain atoms. This behaviour suggests a separation of the contributions of the scaffold and lateral chains atoms to the molecular electronic structure, as highlighted by the comparison of the Molecular Orbitals (MOs) of the parent compound (NSC314622) and of the two derivatives, shown in Fig. S1, S2 and S3. All the MOs from HOMO (H) to LUMO+3 (L+3) are almost identical for the three compounds, and involve only Atomic Orbitals (AO) belonging to the scaffold moiety. Such a correlation is also evident in the H-1 to H-6 MOs, the difference being due only to the presence of the H-2 orbital for NSC725776, and of the H-1 and H-4 orbitals for NSC724998 (located on the lateral chain) indicating that the presence of the propyl group in position 6 does not permit any relevant mixing of the scaffold with the lateral chain MOs.

**Figure 4:**
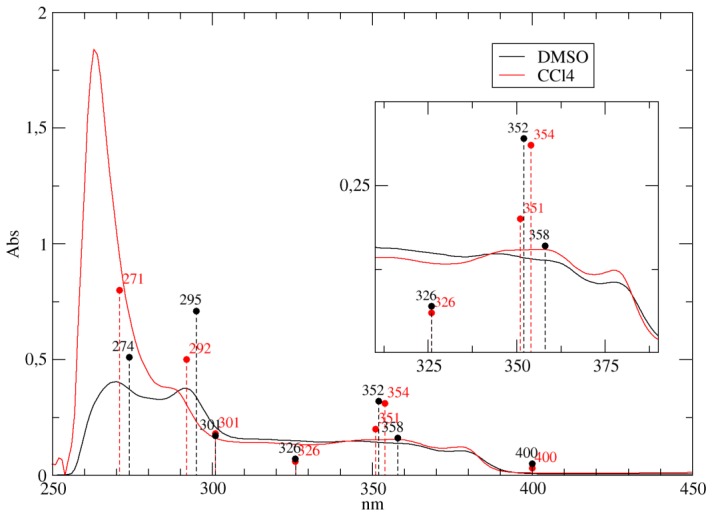
TD-DFT vs. UV-Vis for NSC314622. Comparison of UV/Vis absorption spectra of the indenoisoquinoline NSC314622 in CCl4 (red line) and in DMSO (black line) with TD-DFT calculated transitions. The inset represents a close-up view of the absorption in the 300–400 nm interval. The TD-DFT transitions (dashed vertical lines), calculated in CCl4 and DMSO using C-PCM, are reported as red and black circles respectively and the wavelength of the transitions are annotated. The OS are all rescaled by a factor of 0.25 in order to permit a direct visual comparison with experimental spectra.

**Figure 5:**
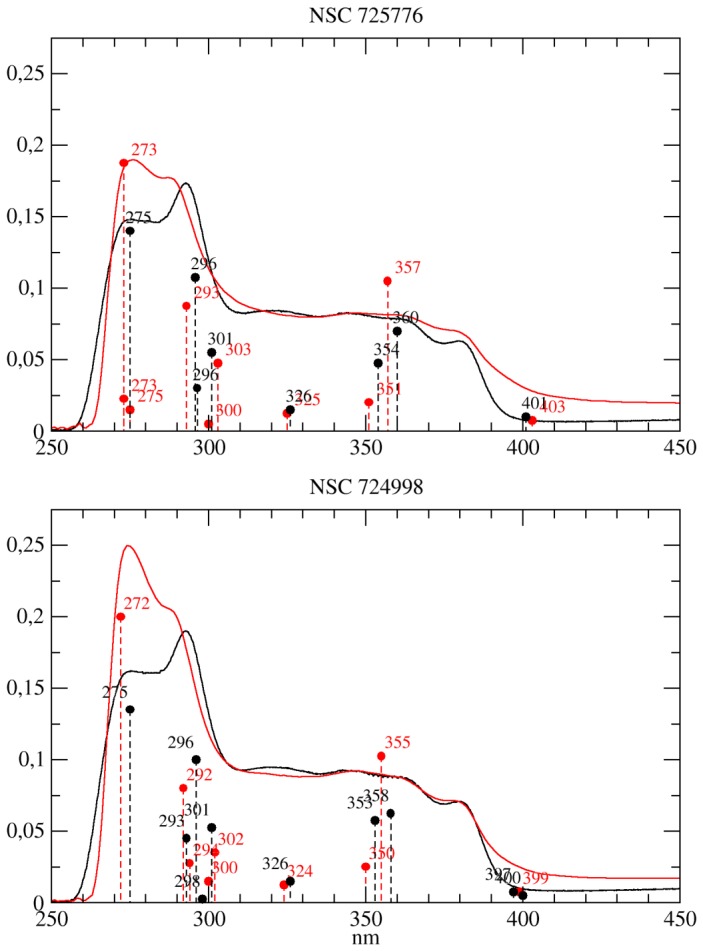
TD-DFT vs. UV-Vis for derivative compounds. Comparison UV/Vis absorption spectra of indenoisoquinoline derivatives NSC725776 (top) and NSC724998 (bottom) in CCl4 (red lines) and in DMSO (black lines) with TD-DFT calculated transitions. The TD-DFT transitions (dashed vertical lines), calculated in CCl4 and DMSO using C-PCM, are reported as red and black circles respectively and the wavelength of the transitions are annotated. The OS are all rescaled by a factor of 0.25 in order to permit a direct visual comparison with experimental spectra.

The electronic transitions of the parent compound and of the two derivatives, calculated at the TD-DFT/B3LYP/6-31+G(d,p) level, having an oscillator strength (OS) > 0.01 in both CCl4 and DMSO, are compared to the experimental bands in Table 1, 2, 3, Fig. 4 and Fig. 5. The two intense experimental bands at 270 and 290 nm, the first one being more intense in CCl4 and the second one in DMSO, are fully reproduced in both intensity and wavelength by TD-DFT computations. In the calculation of NSC314622 in CCl4, a 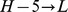 transition at 292 nm and a 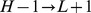 transition at 271 nm are found, with OS 0.5 and 0.8 respectively (Table 1 and Fig. 4 ). The transitions are still present in DMSO and the calculated OS have a trend similar to that experimentally observed, shifting to 0.71 and 0.51, respectively. The corresponding transitions of NSC725776 and NSC724998 at 270 nm (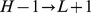 and 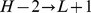 respectively) decrease with a similar trend when passing from CCl4 to DMSO (Table 2, Table 3 and Fig. 5). The NSC725776 compound shows a calculated 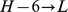 transition at 293 nm (corresponding to the 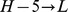 transition of NSC314622) with an increases of the calculated OS from 0.35 to 0.43, when passing from CCl4 to DMSO. In the case of NSC724998 the corresponding 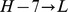 transition, in CCl4, gives rise to two peaks at 294 nm and 292 nm, with OS 0.11 and 0.32 respectively, whereas in DMSO the two bands coalesced into a single transition at 296 nm with an increased OS of 0.40 (Table 3 and Fig. 5). The experimental spectra show a broad absorption band in the range of 305–320 nm, that slightly increases in intensity when passing from CCl4 do DMSO (Fig. 2 and Fig. 3). In this part of the UV-Vis spectra the TD-DFT calculations of NSC314622 reveal the occurrence of the 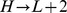 transition at 326 nm, and two transitions (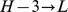 and 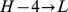) at 301 nm, in both CCl4 and DMSO solvent (Table 1 and Fig. 4). A similar behaviour is observed for NSC72766 and NSC724998 (Table 2, Table 3 and Fig. 5).The experimental solvent-dependent decrease of the intensity of the 354 nm band when passing from CCl4 to DMSO (insert in Fig. 2) is reproduced by the calculated TD-DFT oscillator strength of the corresponding transitions (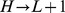 in Table 1, 2, 3).The experimental band at 365 nm is reproduced by an 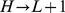 transition, ranging from 354 to 360 nm in the three compounds, while the experimental band at 345 nm is assigned to the 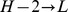 transition for NSC314622 and to the 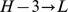 transition for both the NSC725776 and the NSC724998 compound. The experimental 380 nm absorption band can be assigned to the 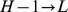 transition at 400 nm for NSC314622 Table 1 and NSC725776 Table 2, corresponding to the 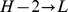 transition for NSC724998 Table 3. In the case of NSC724998 the calculated transition at 400 nm, when passing from CCl4 to DMSO, split in two mixed 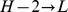 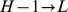 transitions that are not experimentally resolved due to their large broadening. In the visible region of the experimental spectra a very weak absorption is observed (data not shown), whose large band broadening does not permit the identification of a clear peak. The QM calculations in the non-polar CCl4 environment, a weak 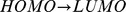 transition at 593 nm, that undergoes a bathochromic shift of 12 nm, when passing into the polar/aprotic DMSO solvent.

**Table 1:** NSC314622 in aprotic solvents.

CCl4	*Exp.* a	DMSO	*Exp.* a	TD-DFT CCl4	TD-DFT DMSO	trans.(HOMO = 95)
				593 (0.02)	605 (0.02)	*H*→*L*
380	sh	380	sh	400 (0.03)	400 (0.05)	*H*−1→*L*
365	w	365	w	354 (0.31)	358 (0.16)	*H*→*L*+1
345	w	345	w	351 (0.2)	352 (0.32)	*H*−2→*L*
				326 (0.06)	326 (0.07)	*H*→*L*+2
310	w	310	w			
					301 (0.17)	*H*−3→*L*
				301 (0.18)		(*H*−4→*L*) + (*H*−3→*L*)
				292 (0.50)	295 (0.71)	*H*−5→*L*
290	str	290	str			
263	v.str	270	str	271 (0.80)	274 (0.51)	*H*−1→*L*+1

Comparison of the TD-DFT transitions of NSC314622, in CCl4 and DMSO, with the experimental UV-Vis absorption peaks, cfr Fig. 2.

a v.str: very strong, str: strong, w: weak, sh: shoulder.

**Table 2:** NSC725776 in aprotic solvents.

CCl4	*Exp.* a	DMSO	*Exp.* a	TD-DFT CCl4	TD-DFT DMSO	trans.(HOMO = 120)
				591 (0.03)	605 (0.02)	*H*→*L*
380	sh	380	sh	403 (0.03)	401 (0.04)	*H*−1→*L*
365	w	365	sh	357 (0.42)	360 (0.28)	*H*→*L*+1
345	w	345	sh	351 (0.08)	354 (0.19)	*H*−3→*L*
				325 (0.05)	326 (0.06)	*H*→*L*+2
315	w	315	sh			
				303 (0.19)	301 (0.22)	(*H*−4→*L*) + (*H*−5→*L*)
				300 (0.02)		(*H*−6→*L*) + (*H*−9→*L*)
					296.4(0.12)	(*H*−7→*L*) + (*H*−8→*L*)
					295.8(0.43)	*H*−6→*L*
289	str	291	str	293 (0.35)		(*H*−7→*L*) + (*H*−6→*L*)
				275 (0.06)		(*H*−2→*L+*1) + (*H*−1→*L+*1)
				273 (0.09)		(*H*−8→*L*) + (*H*−9→*L*)
274	str	274	str	273 (0.75)	275 (0.56)	*H*−1→*L+*1

Comparison of the TD-DFT transitions of NSC725776 compound, in CCl4 and DMSO, with the experimental UV-Vis absorption peaks, cfr. Fig. 3.

a str: strong, w: weak, sh: shoulder.

**Table 3:** NSC724998 in aprotic solvents.

CCl4	*Exp.* a	DMSO	*Exp.* a	TD-DFT CCl4	TD-DFT DMSO	trans.(HOMO = 126)
				589 (0.03)	601 (0.02)	*H*→*L*
					400 (0.02)	(*H*−3→*L*) + (*H*−1→*L*)
380	sh	380	sh	399 (0.03)		*H*−2→*L*
					397 (0.03)	(*H*−1→*L*) + (*H*−2→*L*)
365	w	365	w	355 (0.41)	358 (0.25)	*H*→*L*+1
345	w	345	w	350 (0.10)	353 (0.23)	*H*−3→*L*
				324 (0.05)	326 (0.06)	*H*→*L*+2
315	w	315	w			
				302 (0.14)		(*H*−6→*L*) + (*H*−7→*L*)
					301 (0.21)	(*H*−4→*L*) + (*H*−5→*L*)
				300(0.06)	298 (0.01)	(*H*−8→*L*) + (*H*−4→*L*)
					296 (0.40)	*H*−7→*L*
				294 (0.11)		(*H*−4→*L*) + (*H*−7→*L*)
					293 (0.18)	*H*−7→*L*
289	str	291	str	292 (0.32)		(*H*−8→*L*) + (*H*−7→*L*)
						
274	str	274	str	272 (0.80)	275 (0.54)	*H*−2→*L+*1

Comparison of the TD-DFT transitions of NSC724998 compound, in CCl4 and DMSO, with the experimental UV-Vis absorption peaks, cfr. Fig. 3.

a str: strong, w: weak, sh: shoulder.

It can be concluded that the experimentally observed intensity decrease of the 270 nm band and the increase of the 290 nm band, when passing from CCl4 to DMSO, are quite well reproduced by the TD-DFT calculations for all the three compounds.

**Figure 6:**
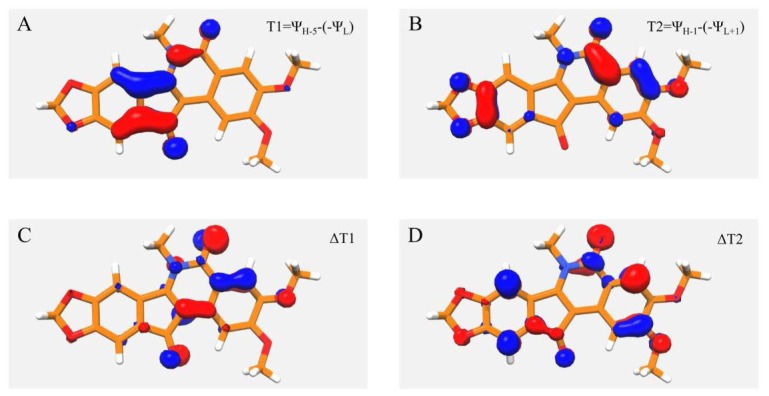
Localization of MOs involved in TD-DFT transitions. A–B) Isodensity surface, enclosing values between −0.05 to +0.05 (red negative values, blue positive values), of the difference of NSC314622 MOs involved in transition at 290 nm (T1) and at 270 nm (T2) (see text for details). C–D) Isodensity surface of the change of T1 and T2 when passing from CCl4 to DMSO. The values of T1 and T2 change range from −0.19 to +0.05. In the Figure are reported the surfaces enclosing the region where the values is between −0.01 and +0.01, in red negative values and blue positive values.

In order to correlate the observed spectra to the molecular electronic structure, a superimposition of the molecular orbitals for the NSC314622 compound involved in the 290 nm (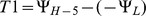) and 270 nm (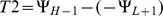) transitions, calculated in DMSO, are shown in Fig. 6A and B respectively. A similar graphical analysis concerning the variation of the wavefunctions involved in a calculated transition has been used on other topoisomerase inhibitors [3]–[5] to localize the molecular regions correlated to the UV-Vis band. The analysis of T1, referring to the calculated transition at 290 nm, reveals the involvement of MOs localized on the lactam group and on the indeno moiety of the molecule (Fig. 6A). In the case of the T2 transition at 270 nm the MOs involved have a much more definite contribution from the oxygen atoms, including the carbonyl oxygen of the lactam group with additional orbitals of the aromatic rings distributed along the whole molecule (Fig. 6B).

A visualization of the variation induced by solvent polarization on the transitions at 290 and 270 nm, when passing from CCl4 to DMSO, can be obtained from the isodensities 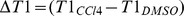 and 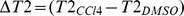 (Fig. 6C and D respectively), calculated as the differences of 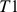 and 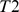 in the two solvents. The figures show that the orbitals of the two carbonyl groups are involved in the solvent dependence for both transitions, indicating that the solvent sensitivity of these groups can be related to the experimentally observed shift. A similar behaviour is found for the other two compounds (data not shown).

### Uv-Vis Spectrum in Aqueous Solvent

**Figure 7:**
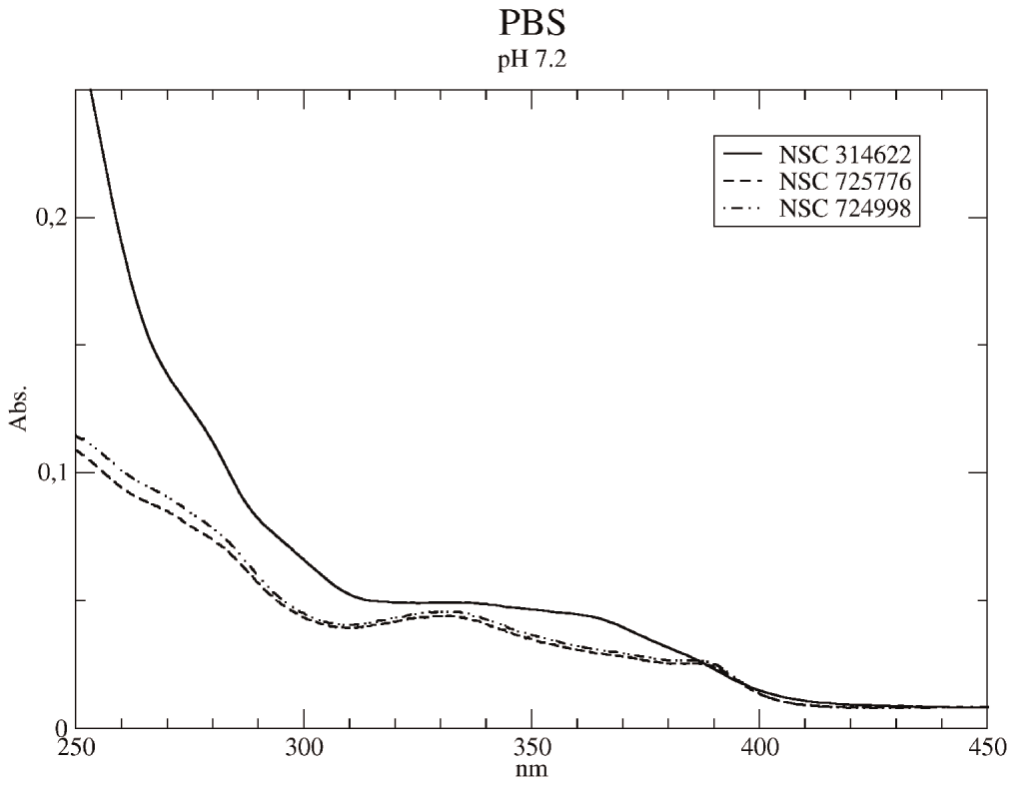
UV-Vis in water. Absorption spectra of NSC314622 (full line), NSC725775 (dashed line) and NSC724998 (dashed dotted line) (10^–5^ M) recorded in PBS at pH 7.2.

The UV-Vis absorption spectra in PBS at pH 7.2 (Fig. 7) are similar for the three compounds and, when compared to the spectra in the non-polar (CCl4) and aprotic (DMSO) solvent, do not show the intense and sharp peaks observed at 290 and 270 nm. In the case of NSC314622 a broad and intense absorption at λ<250 nm overwhelms two shoulders at 270 and 290 nm and, at longer wavelengths, a broad band at 330 nm with a shoulder at 365 nm is found. The two derivative compounds (NSC724988 and NSC725776) show spectra sharing similarities with that of their parental compound, with the presence of a shoulder at 380 nm in place of the one observed at 365 nm (Fig. 7) and with less intense bands in the 250–300 nm region.

The TD-DFT calculations on the NSC314622 compound carried out including the solvent effect through the C-PCM approach (PCM column in Table 4) show transitions at 355–349 nm, 299–294 nm, 272–264 nm and at 238 nm, failing to reproduce the intense experimental absorption at λ<250 nm. This result indicates that the experimental differences observed when passing from CCl4/DMSO to water cannot be accounted for by a simple dielectric effect, but that some direct solvent-induced perturbation must occur on the indenoisoquinoline molecules. Since the experiments on DMSO and CCl4 have indicated that the carbonyl oxygen groups are sensitive to the solvent we have attempted to reproduce the experimental spectrum considering two supramolecular systems composed by the NSC314622 compound in the presence of two explicit water molecules interacting with the carbonyl oxygens called Wat11 and Wat5 (Fig. S4). TD-DFT calculations for the two systems (PCM+Wat5 and PCM+Wat11 in Table 4) doesn’t show any relevant difference when compared with the calculated transitions of the NSC314622 alone using PCM without the explicit water molecules (Table 4). No relevant differences are also observed for a system where the four water molecules are considered at the same time (data not shown).

**Table 4:** NSC314622 in Water (1).

PBS	*Exp.* a	PCM	trans.	PCM+2Wat5	trans.	PCM+2Wat11	trans.
365	sh						
		355 (0.20)	*H*→*L*+1	359 (0.20)	*H*→*L*+1	354 (0.31)	*H*→*L*+1
		349 (0.23)	*H*−2→*L*	352 (0.24)	*H*−1→*L*		
330	w						
		299 (0.14)	*H*−3→*L*			308 (0.31)	*H*−3→*L*
290	m	294 (0.72)	*H*−5→*L*	294 (0.79)	*H*−5→*L*		
270	m	272 (0.46)	*H*−1→*L+*1	277 (0.46)	*H*−1→*L+*1	274 (0.52)	*H*−1→*L+*1
		264 (0.14)	*H*→*L*+3	266 (0.26)	*H*−2→*L+*1		
250	str						
		238 (0.16)	*H*−2→*L+*2	238 (0.13)	*H*−2→*L+*2	239 (0.16)	*H*−2→*L+*2

Comparison of the experimental peaks of the UV-Vis spectrum of NSC314622 in PBS with the TD-DFT transitions, for the compound in water (PCM) and for the compound having two explicit water molecules in position 5 (PCM+2Wat5) or in position 11 (PCM+2Wat11).

a str: strong, w: weak, sh: shoulder.

**Figure 8:**
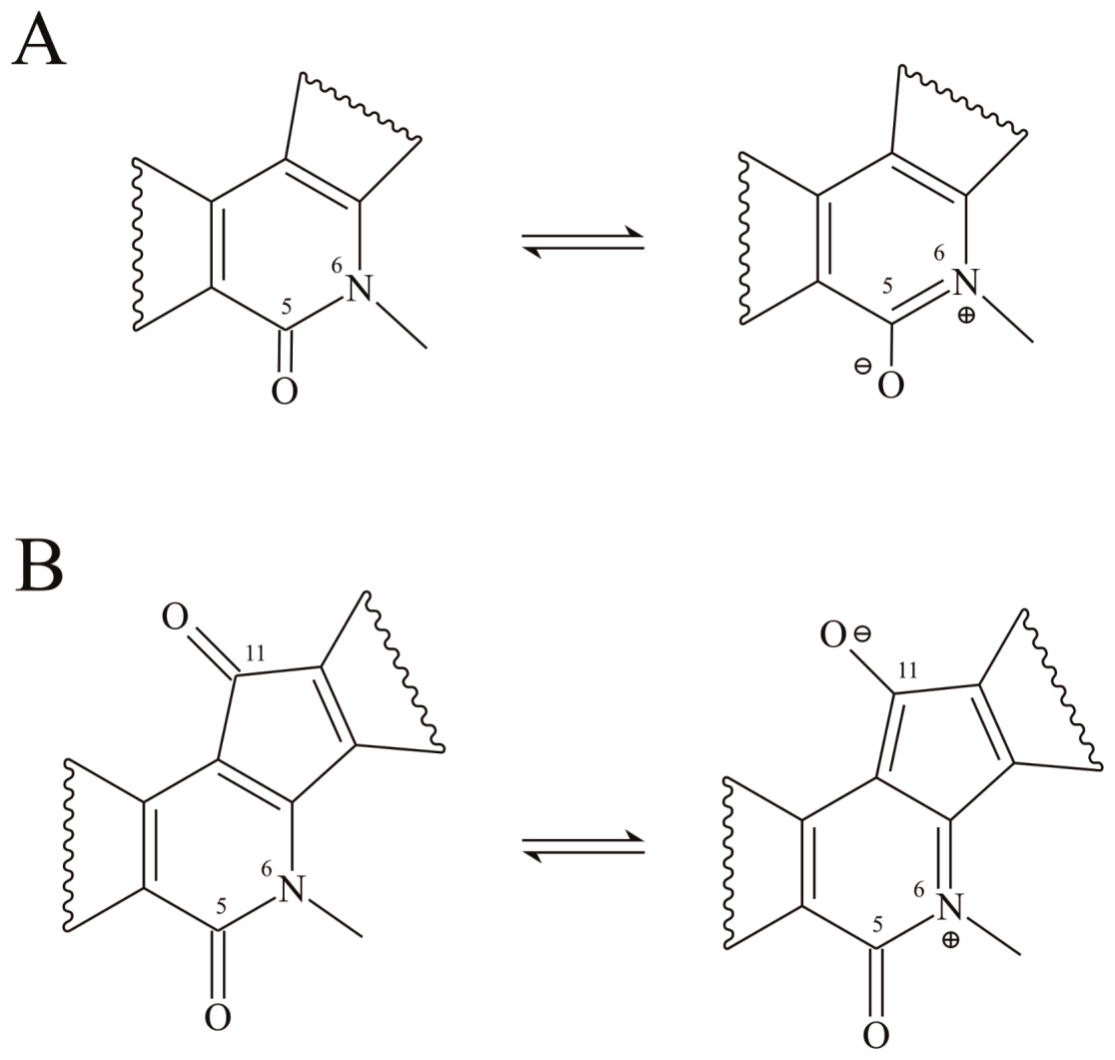
IQN in equilibrium with its zwitterionic forms. A) polarization of the lactam group B) polarization of the carbonyl group of the indeno moiety.

The polarization effect on the carbonyl groups (Fig. 8) has been then taken into account using two forms singly protonated on the keto oxygens in position 5 and 11, called P5 and P11 (Fig. S4) [29] and a form protonated in both positions, called P5+P11. The doubly protonated, and the singly protonated forms have relatively intense transitions at 250 nm (Table 5). In detail the P5+P11 form shows an 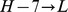 transition at 253 nm (OS 0.17), and an 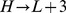 transition at 250 nm (0.13), the P11 form shows an 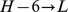 transition at 249 nm (0.26), an 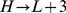 transition at 245 nm (0.16) and an 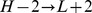, at 238 nm, while the P5 form shows an 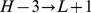 transition at 262 nm (0.14). The other UV/Vis experimental features are also reproduced by the protonated forms. In detail the P5 form reproduces the two shoulders at 270 and 290 nm the broad experimental band at 330 nm is reproduced by either the P11 and the P5+P11 forms, while the 365 nm shoulder is reproduced by the P5 and the P5+P11 forms (Table 5). The experimental spectrum in water can then be reproduced by a combination of the neutral and the protonated forms of IQN. The IQN protonated forms, must be considered as an extremal model for the stabilization of the polarized forms likely induced by hydrogen bond interactions of the keto oxygens (Fig. 8). The calculation then provides strong evidence of the high polarizability of the two carbonyl groups that represent useful reporters of the micro-environment solvatation state.

**Table 5:** NSC314622 in Water (2).

PBS	*Exp.* a	PCM+P5	trans.	PCM+P11	trans.	PCM+P5+P11	trans.
						555 (0.14)	*H*−1→*L*
		377 (0.41)	*H*−1→*L*			387 (0.19)	*H*→*L*+1
365	sh						
		360 (0.1)	*H*−2→*L*			360 (0.27)	*H*−3→*L*
330	w			334 (0.59)	*H*−3→*L*	333 (0.21)	*H*−4→*L*
				308 (0.84)	*H*→*L*+2		
						315 (0.1)	*H*−1→*L+*1
						296 (0.9)	*H*→*L*+2
290	m	292 (0.46)	*H*→*L*+2	277 (0.46)	*H*−1→*L+*1	274 (0.52)	*H*−1→*L+*1
		281 (0.94)	*H*−2→*L+*1	281 (0.51)	*H*−1→*L+*1	284 (0.36)	*H*−1→*L+*1
		273 (0.12)	*H*−3→*L*				
270	m						
		262 (0.14)	*H*−3→*L+*1				
						253 (0.17)	*H*−7→*L*
250	str			245 (0.16)	*H*→*L*+3	250 (0.13)	*H*→*L*+3
				249 (0.26)	*H*−6→*L*		
				238 (0.16)	*H*−1→*L+*2		

Comparison of the experimental peaks of the UV-Vis spectrum of NSC314622 in PBS with the TD-DFT transitions, for the compound in water, singly protonated in position 5 (PCM+P5), in position 11 (PCM+P11) or doubly protonated (PCM+P5+P11).

a str: strong, w: weak, sh: shoulder.

## Discussion and Conclusions

The UV-Vis spectroscopic signatures of three compounds belonging to the IQN family (Fig. 1) have been systematically investigated using a combined computational procedure and the results have been compared to the experimental spectrum in CCl4 and DMSO. TD-DFT calculations provide a coherent picture of the IQN behaviour, confirmed by the close agreement of the computed and experimental UV-Vis spectra in the two solvents. In detail the decrease of 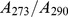 intensity ratio, when passing from CCl4 to DMSO (Fig. 2, Fig. 3), is reproduced by the calculations showing that this can be related to the solvent polarity sensitivity of the keto-oxygens in position 5 and 11 of the molecular scaffold (Table 1 2 3 and Fig. 4 5 and 8).

The experimental UV-Vis spectrum undergoes dramatic changes, such as the disappearance of the 290 and 270 nm peaks and the appearance of an intense peak at λ<250 nm, when recorded in PBS (Fig. 7). Such a spectroscopic feature cannot be reproduced including the solvent effect through C-PCM and not even by considering the presence of explicit water molecules proximal to the keto oxygens in position 5 and 11 (Table 4), at variance with results recently found for camptothecins where the overall spectral features have been reproduced considering the contribution of different drug populations having defined water distribution around them [3]–[5]. In the case of IQNs it has been necessary to model the system considering polarized configurations with a partial negative charge localized on the two keto-oxygens and a positive charge on the nitrogen in position 6 (Fig. 8). This modelling is necessary because the presence of the two keto-oxygens on the central rings determines a ground state composed by several contributing structures having a high degree of polarization, that are likely stabilised by protic interactions. A similar behaviour has been observed in an isoquinolinone derivative (2-methyl-1,4-diphenylbenzo[g]isoquinolin-3(2 H)-one, MDP), where the aromatic tautomer (cfr. Fig. 8A) is stabilized by solvent hydrogen-bond donors [29] and TD-DFT calculations of MDP having the keto-oxygen protonated, explain the experimental blue-shift of fluorescence emission in terms of a stabilization of the zwitterionic form by solvent hydrogen bond. In our case, the various protonated forms of IQN (P5, P11 and P5+P11 in Fig. S4) taken as extreme models of the polarized configurations, likely stabilized by hydrogen bond interaction with a water molecule in the “real” system, show calculated transitions around 250 nm that are not found in the calculations performed on the deprotonated form using only the C-PCM model (Table 4). In detail the polarization involving the oxygen in position 11 plays a major role in the appearance of the absorption band at λ<250 nm.

Our coupled experimental and computational chemistry approach indicates that the region 250–290 nm of the UV absorption spectrum of indenoisoquinoline (NSC314662) and of two clinically relevant derivatives (NSC724998 and NSC725776) is highly sensitive to the environment polarity. A sensitivity to the solvent polarity could likely be expected, but the TD-DFT calculations permits to attribute such a dependence to the keto-oxygens in position 5 and 11, that can undergo a polarization stabilized by protic interaction with the solvatation micro-environment. As a matter of fact the X-ray structures of the ternary complexes in presence of IQN derivatives show that the keto-oxygens are involved in the formation of an hydrogen bond with Arg364 residue of Top1 [10], [11], suggesting that the IQNs UV-Vis spectrum can be used as a fingerprint for the investigation of the interaction of the drug with its biomolecular target. Titration of the Topo1/DNA complex with indenoisoquiniline following the UV/Vis spectrum modification is far beyond the scope of this work, that is aimed to the identification of chemical groups sensitive to the environment through a coupled spectroscopic and computational approach. The results of the here presented work are in line with other studies, which demonstrated the importance of fluctuations, induced by solvatation on molecular configuration and electronic structure, in the interpretation of UV-Vis spectrum of small molecules [30], [31]. Calculations of the absorption spectrum of the drug in the protein-DNA micro-environment, using more extended sampling of solvatation entropy (e.g. throug ab-initio MD or PMM [32]), represent the next step to identify the spectral features that would permit a direct experimental evaluation of the drug binding to the Topo1-DNA complex through UV/Vis absorption.

## Materials and Methods

### UV-Vis Spectra

NSC314622, NSC724998 and NSC725776 (Fig. 1B) were prepared as previously described [20]. Powders have been dissolved in DMSO to form stock solutions at a concentration of 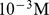. Stocks have been diluted in 10 mL of CCl4, DMSO and phosphate buffered saline (PBS, pH 7.2) to a final concentration of 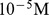, and the UV-Vis spectra have been recorded with a Perkin-Elmer UV-Vis Lambda 2 spectrophotometer in the range 250–450 nm.

### Computational Chemistry

All calculations have been performed using the Gaussian03 package [33], on the “Matrix” cluster at CASPUR, using an increasing level of complexity for the description of the molecular electronic state. Starting molecular geometries for the three compounds in Fig. 1B have been built using Molden4.7 [34], and the minimum energy structures, in the singlet state, have been fully optimized at the RHF/3-21G level in gas phase and then in non-polar (CCl4), polar aprotic (DMSO) and polar protic (water) environment, including solvent effects by means of the COSMO variant of the polarizable continuum model (PCM) [35]. Correlation effects have been taken into account via density functional theory (DFT) performing a full geometrical optimization using the B3LYP hybrid functional [36], [37] and the 6-31+G(d,p) basis set. The Wat5 and Wat11 supra-molecular system composed by the NSC314622 compound and two explicit water molecules coordinated in position 5 and 11 respectively (see Fig. S4), have been obtained starting from the PCM (water) optimized structure, by further relaxing the molecular geometry. The geometries of P5 and P11 configurations in Fig. S4 and the doubly protonated form, where the keto-oxygens in position 5 and 11 are replaced by a hydoxyl group, has been obtained following a full geometrical relaxation performed restricting the total charge of the system to +1. The electronic transition of the three compounds in the three solvents, and the various configurations in Fig. S4 in water, have been obtained using TD-DFT calculations [38] on the corresponding optimized geometries using the same basis set of reference for these computations (6-31+G(d,p)).

## Supporting Information

Figure S1 **Comparison of the LUMO+3 to LUMO molecular orbitals calculated at the B3LYP/6-31+G(d,p) level for NSC314622,NSC725776 and NSC724998.**
(EPS)

Figure S2 **Comparison of the HOMO to H-2 molecular orbitals calculated at the B3LYP/6-31+G(d,p) level for NSC314622 with HOMO, H-1 and H-3 of NSC725776 and HOMO, H-2 and H-3 of NSC724998.**
(EPS)

Figure S3 **Comparison of the H-3 to H-6 molecular orbitals calculated at the B3LYP/6-31+G(d,p) level for NSC314622 with H-4 to H-7 for NSC725776 and H-5 to H-8 for NSC724998.**
(EPS)

Figure S4 **Molecular configuration of NSC314622 singly protonated in position 5 and 11 (P5 and P11) and of two supramolecular systems composed by NSC314622 and 2 explicit water molecules coordinated at the keto-oxygens in position 5 and 11 (2Wat5 and 2Wat11).**
(EPS)

Coordinates S1 **Optimized cartesian coordinates of Indenoisoquinolines discussed in the article.**
(ZIP)
